# The Impact of Redox, Hydrolysis and Dehydration Chemistry on the Structural and Magnetic Properties of Magnetoferritin Prepared in Variable Thermal Conditions

**DOI:** 10.3390/molecules26226960

**Published:** 2021-11-18

**Authors:** Lucia Balejčíková, Karel Saksl, Jozef Kováč, Anne Martel, Vasil M. Garamus, Mikhail V. Avdeev, Viktor I. Petrenko, László Almásy, Peter Kopčanský

**Affiliations:** 1 Institute of Hydrology SAS, Dúbravská Cesta 9, 84104 Bratislava, Slovakia; 2 Institute of Materials Research Slovak Academy of Sciences, Watsonova 47, 04001 Košice, Slovakia; ksaksl@saske.sk; 3 Institute of Experimental Physic SAS, Watsonova 47, 04001 Košice, Slovakia; jkovac@saske.sk (J.K.); kopcan@saske.sk (P.K.); 4 Institut Laue-Langevin, 71 Avenue des Martyrs, 38042 Grenoble, France; martela@ill.fr; 5 Helmholtz-Zentrum Hereon, Max-Planck-Street 1, 21502 Geesthacht, Germany; 6 Joint Institute for Nuclear Research, Joliot-Curie Str. 6, 141980 Dubna, Russia; avd@nf.jinr.ru; 7 BCMaterials, Basque Centre for Materials, Applications and Nanostructures, 48940 Leioa, Spain; viktor.petrenko@bcmaterials.net; 8 IKERBASQUE, Basque Foundation for Science, 48009 Bilbao, Spain; 9 Institute for Energy Security and Environmental Safety, Centre for Energy Research, Konkoly Thege Mi-klos ut 29-33, 1121 Budapest, Hungary; almasy.laszlo@ek-cer.hu

**Keywords:** magnetoferritin, magnetite, loading factor, protein stability, aqueous medium

## Abstract

Ferritin, a spherically shaped protein complex, is responsible for iron storage in bacteria, plants, animals, and humans. Various ferritin iron core compositions in organisms are associated with specific living requirements, health state, and different biochemical roles of ferritin isomers. Magnetoferritin, a synthetic ferritin derivative, serves as an artificial model system of unusual iron phase structures found in humans. We present the results of a complex structural study of magnetoferritins prepared by controlled in vitro synthesis. Using various complementary methods, it was observed that manipulation of the synthesis technology can improve the physicochemical parameters of the system, which is useful in applications. Thus, a higher synthesis temperature leads to an increase in magnetization due to the formation of the magnetite phase. An increase in the iron loading factor has a more pronounced impact on the protein shell structure in comparison with the pH of the aqueous medium. On the other hand, a higher loading factor at physiological temperature enhances the formation of an amorphous phase instead of magnetite crystallization. It was confirmed that the iron-overloading effect alone (observed during pathological events) cannot contribute to the formation of magnetite.

## 1. Introduction

Ferritin, since its first isolation in 1937 by Vilém Laufberger [1], is still an interesting research object. This metalloprotein is especially applicable in medicine as nanocarriers [2,3,4,5] or nanoplatforms for inflammation detection [6]. Its structural variations and metabolic roles in living systems (physiological as well as pathological significance) are another reason for intensive studies of this nanomaterial [7].

Ferritin is a spherical cage that is 12 nm in diameter, composed of an apoferritin shell, which surrounds ferrihydrite (FeOOH) nanocrystals [8]. Meldrum et al. demonstrated in 1992 the use of apoferritin as a reaction environment for the in vitro synthesis of iron oxides (*γ*-Fe_2_O_3_/Fe_3_O_4_) [9]. The novel material called “magnetoferritin” gained wide recognition for its application possibilities in bionanotechnology (separation, biosensors, nanodevices) or biomedicine (hyperthermia therapy, cell imaging, MRI diagnostics, targeted drug delivery) [10,11,12]. The SARS-CoV-2 virus infection has led to expanding ferritin cage application. Ferritin functionalized by the SARS-CoV-2 receptor-binding domain [13], or spike protein [14] could be an advantageous approach to prevent the spread of COVID-19. Similarly, the use of magnetoferritin in such a vaccine design could increase its visibility in MRI [15,16]. A great advantage of iron oxide nanoparticles inside magnetoferritin could be an increase in the efficiency of radiation therapy due to their ability to produce reactive oxygen species [17].

In parallel, in 1992, Kirschwink et al. observed magnetite nanocrystals in the brain of patients with neurodegenerative diseases [18]. The presence of magnetite was also confirmed in tumor tissues [19]. The expected precursor for the synthesis of magnetite in vivo might be the ferritin iron core [19,20] followed by the subsequent formation of “biogenic magnetoferritin”. All factors and metabolic pathways that affected the in vivo crystallization of this mineral have not been fully revealed. A combination of iron overloading, temperature, pressure, pH value, magnetism, electromagnetic radiation, reaction kinetics, the presence of various molecules, oxidants/reductants (flavonoids, vitamins, superoxide, Aβ peptide) [21,22,23,24,25], metabolic imbalances, genetics (transcription, translation or folding deficiencies), structural or functional disorders, and the role of short- and long-term interactions (Levinthal paradox) [26] was suggested. Simple magnetotactic bacteria, *Magnetospirillum magnetotacticum*, can naturally form highly monodisperse single-domain magnetite nanocrystals [27]. Birds are equipped with similar magnetite nanocrystals functioning as sensitive indicators for orientation in the Earth’s geomagnetic field [28]. Difficulties in the simulation of physiological mechanism for magnetite formation in vitro are related to incomplete understanding of the biochemical complexity of each organism, including its health state. Commonly used laboratory procedures for synthesis of magnetite nanoparticles include high temperatures (50–80 °C) and alkaline conditions with pH > 8, based on a co-precipitation method with a precisely pre-established ratio of Fe^2+^ and Fe^3+^ ions in aqueous solution. Along with it, the investigation of intermediate phases formed during the reaction pathways, showed the sensitivity of the chemical composition, magnetic properties, size distribution and crystallinity of nanoparticles to the physicochemical properties of the surrounding liquid medium [29].

Magnetoferritin can be considered as a suitable model system for in vitro studies of magnetite bio-mineralization in a constrained, uniformly sized hollow cavity. Magnetic nanoparticles prepared at 65 °C, alkaline pH, constant stirring and controlled addition of Fe/oxidant into the apoferritin nano-cage, were characterized in previous studies. The number of iron atoms added to the reaction affected the size of the iron core, the formation of aggregates, and the chemical composition [30,31,32].

Our research aimed at revealing the effect of physicochemical properties of the aqueous medium (iron loading, pH, and synthesis temperature) on the organic and inorganic magnetoferritin model structure using ultraviolet and visible spectroscopy, dynamic light scattering, zeta potential measurements, SQUID magnetometry, magneto-optical, cryogenic transmission electron microscopy, X-ray diffraction and small-angle neutron scattering methods. These studies clarify to what extent the structure of magnetoferritin can be optimized regarding the magnetite loading and structural stability.

## 2. Results and Discussion

### 2.1. Formation of Iron Cores inside Apoferritin In Vitro and Characterization of Magnetoferritin

The globular protein shell of apoferritin consists of 24 subunits. The relative molecular weight of ~450 kDa depends on the organism species, as well as the type of source tissue. The interaction of two functional and genetically different H- and L-subunits labeled in accordance with their molecular weight (H-heavy ~21 kDa and L-light ~19 kDa) allows packing of a typical heteropolymeric protein. The predominance of α-helical secondary structures ensures protein (un)folding and flexibility. The subunits are arranged in 12 sets of anti-parallel pairs with 4-, 3-, and 2- axes of symmetry. At the intersection of these axes, there are eight 3-fold hydrophilic and six 4-fold hydrophobic intersubunit channels with a diameter of 4 Å for the transport of iron ions and electrons. H-subunits exhibit natural ferroxidase activity essential for the uptake and storage of iron. L-subunits are necessary for the mineralization of iron inside the (apo)ferritin cavity. The distribution ratio of H-/L- subunits is tissue specific. Up to 70% of H- subunits can be found in tissues with high oxidation rates (i.e., in the heart and brain), and 90% of L- subunits are in tissues that perform mainly the storage function and experience much less oxidative stress (i.e., spleen and liver) [8]. In this study, we use horse spleen (apo)ferritin due to its high degree and quality of purification.

Samples of aqueous magnetoferritin (MFer) with seven different loading factors (LFs), defined as the average number of iron atoms per one protein cage, were prepared. After the procedure of in vitro iron incorporation into the apoferritin shell, UV-Vis absorbance was used to calculate the iron-to-protein ratio and determine LF. LFs for the prepared samples with average errors of less than 2% are summarized in Table 1.

The hydrodynamic diameter, D_HYDR_ (Table 1) was determined using dynamic light scattering (DLS). D_HYDR_ corresponds to the effective size of the biomacromolecule and is highly dependent on the ionic strength, particle concentration, and temperature. The D_HYDR_ values collected in Table 1 indicate the predominance of polydisperse MFer aggregates. Agglomerates of iron-protein particles contribute to higher D_HYDR_ compared to the theoretical diameter of the apoferritin molecule of 12 nm [8]. The non-linear dependence of D_HYDR_ on LF was caused most likely by various temperature conditions during synthesis aimed at obtaining various types of iron compounds. The polydispersity index (PDI) confirms the heterogeneity of the samples (Table 1). Generally, PDI values below 0.7 are accepted for relatively stable nano-colloidal systems, suitable for applications in pharmaceutical practice.

**Figure 1:**
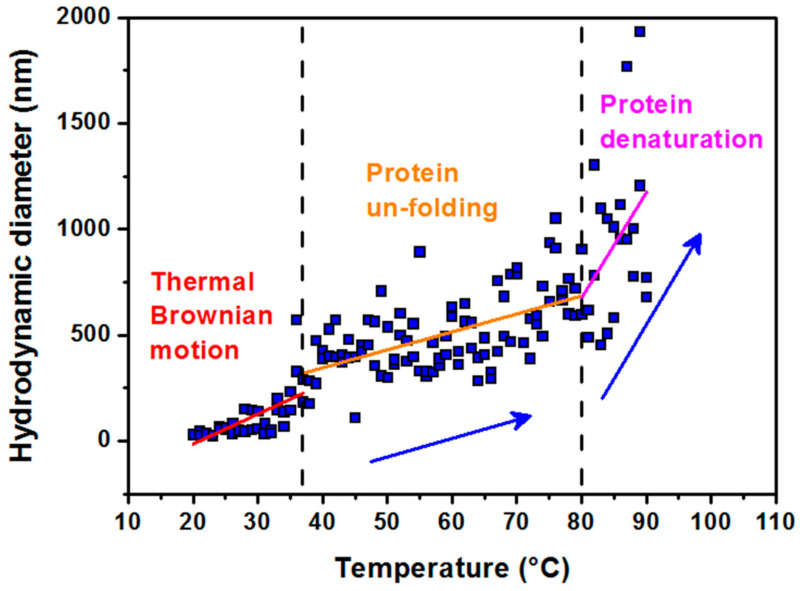
Hydrodynamic diameter dependence vs. temperature of the solutions. Data were obtained in the range from 20 to 90 °C for a colloidal solution of apoferritin in AMPSO buffer at pH 8.6, showing three characteristic modes of apoferritin conformation. The first region of 20–37 °C (physiological temperature) refers to the thermal Brownian motion of apoferritin particles. The second region of 37–80 °C shows the protein unfolding behavior and the third one above 80–90 °C illustrates the protein denaturation region.

Due to the fact that laboratory crystallization of magnetite in an aqueous medium requires higher temperatures [23], we focused on the analysis of thermal stability of the protein shell, apoferritin, used for synthesis of MFer. The characteristic size was monitored by DLS during heating from 20 to 90 °C (Figure 1).

A stable size is observed in the range from 20 °C to a physiological temperature of 37 °C. A slight increase in size with temperature is most likely a consequence of the thermal Brownian motion. The second region up to 80 °C, which reflects the denaturation (melting) point, is indicative of a slow unfolding of the quaternary protein structure with the preservation of the secondary structure (α-helices) [33]. The melting point is the transition temperature at which the concentration of the denatured protein is equal to the concentration of the native globular protein. Rapid hydrodynamic size growth above 80 °C signifies protein denaturation. The results of our previous study based on small-angle scattering data showed thermal decomposition of the empty apoferritin shell exposed to a standard synthesis temperature of 65 °C for 100 min [34]. It should be mentioned that apoferritin, used as a reaction environment for synthesis of MFer, relies on the self-assembly ability of the protein shell [3,4,5,35]. Minimizing the contacts of hydrophobic amino acids with water molecules is the driving force behind the packing process. Thermal dissolution of independent protein subunits in a specific aqueous medium could be helpful for reversible folding processes during MFer synthesis within the “unfolded region” marked in Figure 1. Based on this consideration, a MFer colloidal solution can contain single particles, partially unfolded structures, or independent subunits together with a population of protein agglomerates. DLS measures the average of this heterogeneous population in the aqueous sample. In Table 1, this heterogeneous state corresponds to MFer LF 447 prepared at a temperature close to the melting point of 75 °C. A similar effect is observed for the MFer LF 559 sample prepared at a physiological temperature of 37 °C, where the growth of D_HYDR_ could be due to the addition of Mohr′s salt, causing the formation of agglomerates (i.e. “salting out” effect). Lower D_HYDR_ values determined between 14–20 nm (Table 1) refer to MFer nanoparticles prepared using a standard synthesis procedure.

The zeta potential values (Table 1) indicate good colloidal stability of all samples. The total charge belongs to the dominant hydrophilic negatively charged amino-acid residues on the surface layer contributing to the good solubility of the protein in water, which is necessary for biological applications. Different values of the zeta potential are related to the variation in pH, ionic strength, particle concentration, and viscosity of aqueous samples, which could be influenced by changes in LF and the type of the iron core. The zeta potential for apoferritin and ferritin was lower compared to MFer. The increase in parameters for MFer is most likely due to the rearrangement of protein chains during synthesis.

**Figure 2:**
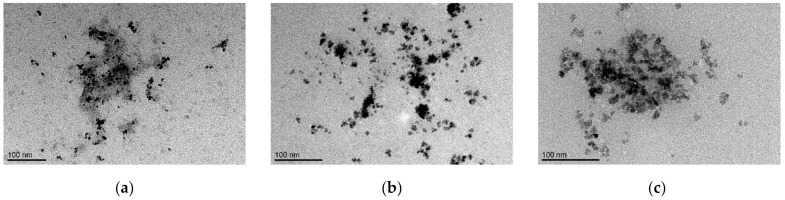
Cryogenic transmission electron microscopy images of magnetoferritin nanoparticles. Loading factor of magnetoferritin: (**a**) 160; (**b**) 490; (**c**) 510.

The morphology of prepared samples was studied using cryogenic transmission electron microscopy (cryoTEM). Illustrative cryoTEM images (Figure 2) show the quasi-spheroidal shape of individual nanocrystals of MFer samples with a diameter of about ~10 nm. The presence of aggregates ~30–50 nm in size indicates sample polydispersity, most likely induced by dipole-dipole interactions of magnetic nanoparticles, as well as the presence of partially un-folded protein structures. The observed large objects are normally formed when drying samples on a grid, where particles interact after removing water.

### 2.2. Synthesis Temperature Impact on the Magnetic Properties of Magnetoferritin

**Figure 3:**
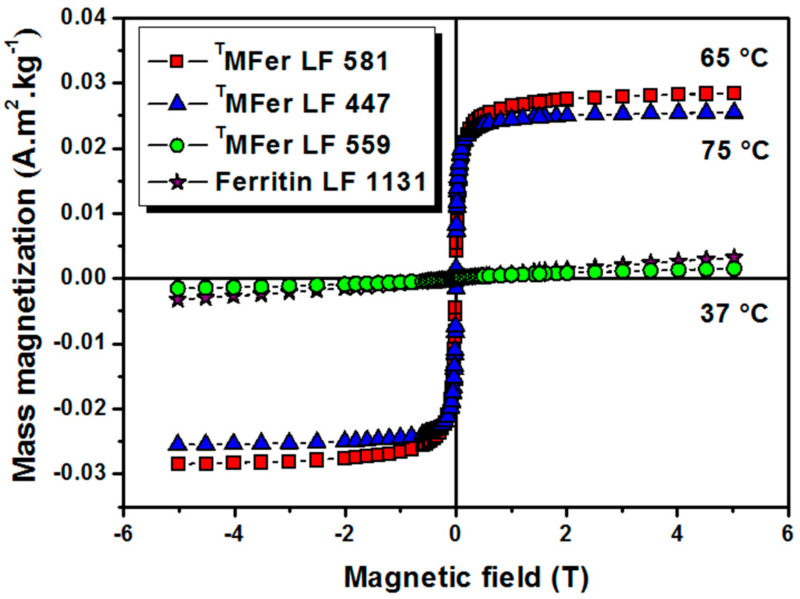
Magnetization curves of magnetoferritin. Data were obtained at 22 °C for ^T^MFer (magnetoferritin) with LF (loading factor): 581, 447, and 559 prepared at various synthesis temperatures marked on the graph (37, 75 and 65 °C). T prefix in ^T^MFer labeling was used for specification of magnetoferritin samples prepared at three different synthesis temperatures with similar LF.

To investigate the effect of the synthesis temperature on the magnetic properties, MFer samples with similar LF of about 500 were prepared at different synthesis temperatures. The proper *LF* ~500 was chosen to achieve stable samples and a measurable size-dependent signal from the core. Magnetization vs. magnetic field dependences of ^T^MFer samples with similar LFs of 447, 559, and 581, prepared at synthesis temperatures of 75 °C, 37 °C, and 65 °C respectively, did not show any hysteresis (Figure 3). ^T^MFer samples with LF 581 and 447 show saturation in contrast to ^T^MFer LF 559 with a linear dependence of magnetization on magnetic field, similar to ferritin. The increase in magnetization is associated with higher synthesis temperatures (65 °C and 75 °C) that enhance the probability of formation of magnetite nanoparticles. 

**Figure 4:**
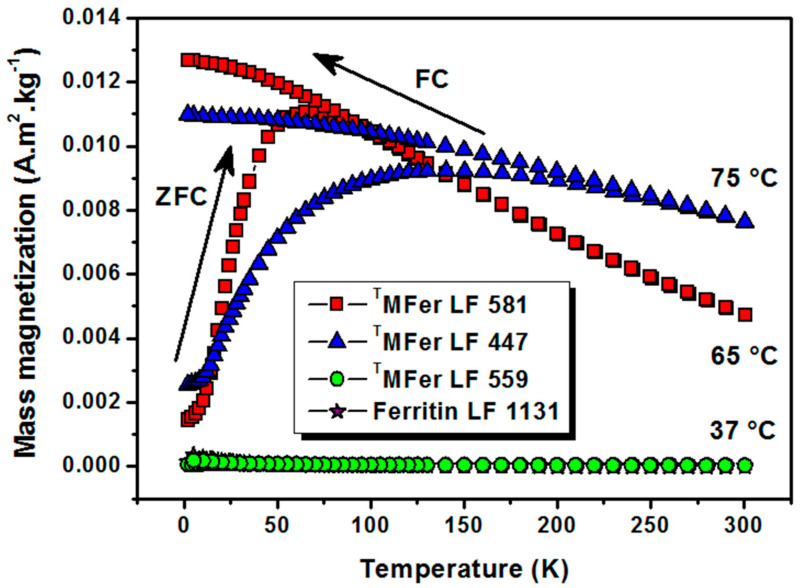
Thermomagnetic curves after cooling at zero magnetic field (ZFC)/under 7.96 kA/m magnetic field (FC) for magnetoferritin. ^T^MFer with LF: 581, 447 and 559 was prepared at various synthesis temperatures marked on the graph (37, 65, and 75 °C). T prefix in ^T^MFer labeling was used for specification of magnetoferritin samples prepared at three different synthesis temperatures with similar loading factor (LF).

The thermomagnetic curves (Figure 4) measured after cooling the powder ^T^MFer sample in zero magnetic field (ZFC) and under an applied magnetic field (FC) of 7.96 kA/m showed a blocking temperature T_B_ of about 55 K (i.e., −218.15 °C) for LF 581. The curve shape for ^T^MFer with LF 447 does not allow determining T_B_, most likely due to the presence of aggregates. At low temperatures, the thermal energy decreases and magnetic moments should become blocked. We assume that the zero magnetic moment of the system in the FC-mode was hard to achieve most likely because of the presence of larger aggregates, which contributed to the broad shape of the curve. A gradual increase in T_B_ was previously observed when magnetic nanoparticles were surface-functionalized [36].

The protein aggregation (Table 1) and observed partial destruction of the shell [34] lead to clustering of the magnetic nanoparticles. This phenomenon causes an increase of the magnetic moment, and, together with the nanoparticle polydispersity, leads to a shift in the T_B_ and broadening of the shape of the ZFC curve. The superparamagnetic character of the sample is partially lost. The character of the thermomagnetic curve supports this assumption and, therefore, the T_B_ can no longer be unambiguously determined [37]. The linear dependence of magnetization on the applied magnetic field (Figure 3), as well as the thermomagnetic curves (Figure 4) of ^T^MFer samples LF 559 and LF 1131 clearly showed paramagnetic behavior.

### 2.3. Synthesis Temperature Impact on the Crystallinity of Magnetoferritin

**Figure 5:**
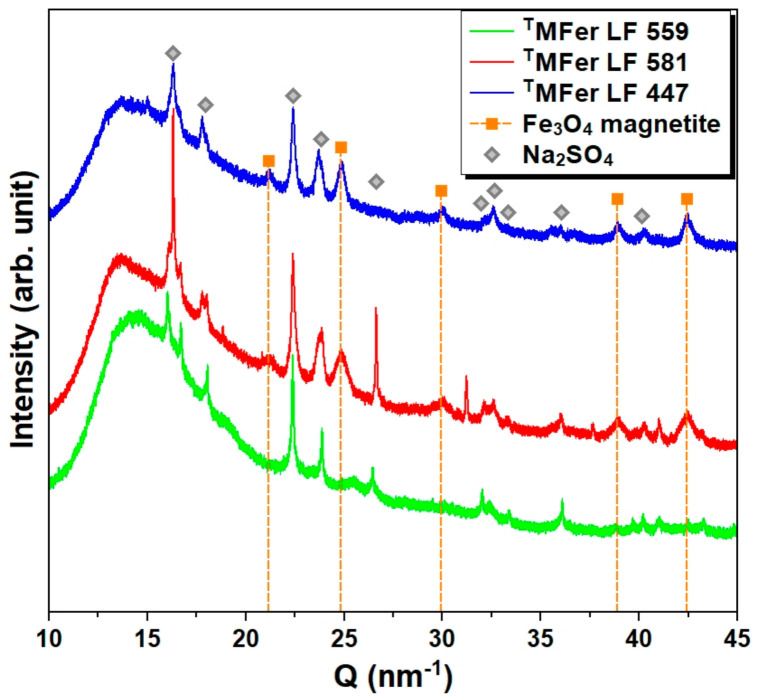
X-ray diffraction patterns for powdered magnetoferritin. ^T^MFer with LF 559, 581, and 447 was prepared at various synthesis temperatures (37, 65, and 75 °C). Marked peaks corresponded to magnetite and Na_2_SO_4_ as major crystalline phases. T prefix in ^T^MFer labeling was used for specification of magnetoferritin samples prepared at three different synthesis temperatures with similar loading factor (LF).

Along with the Bragg peaks from the crystalline phases, X-ray diffraction patterns of ^T^MFer samples show a high proportion of the disordered (non-crystalline) phase, which manifests itself in a strong isotropic background (Figure 5). This amorphous phase could belong to the protein, organic buffer, or reaction products. Protein crystallization is a complex process that requires high purity of samples (removal of reactants and intermediates) and the use of, for example, exchange chromatography [38].

By the method of phase analysis based on X-ray diffraction data, we identified the crystalline phase of sodium sulfate Na_2_SO_4_ in all studied samples, resulted, as we suppose, from the Mohr’s salt reaction with NaOH. Mohr’s salt was used as a source of ferrous ions and NaOH to regulate the pH of the AMPSO buffer. Except for Na_2_SO_4_ crystallization in all samples, the magnetite (Fe_3_O_4_) phase was identified in ^T^MFer samples with LF 581 and 447. This phase is not present in the ^T^MFer sample with LF 559, prepared at the lowest synthesis temperature, i.e., 37 °C (Figure 5), resulting in the non-crystalline phase of the iron core and an increase in the amorphous background.

So far, the iron phases we have determined do not fully correspond to the generally accepted chemical Equation (1) for magnetite synthesis inside apoferritin at 65 °C and pH 8.6, suggested by Wong et al. [30]:

3Fe^2+^ + 2(CH_2_)_3_NO + 4H_2_O → Fe_3_O_4_ + 2(CH_2_)_3_N + 6H^+^ + H_2_O_2._ (1)

The amount of iron ions in aqueous solutions is related to the redox potential (*E_H_*), especially depending on temperature and pH. Dissolved ferrous ions at pH 8.6 accompany hydrated ferrous complexes and the formation of soluble ferrous hydroxides (known as green rust):

2[Fe(H_2_O)_6_]^2+^ + 2H_2_O → [(H_2_O)_4_Fe(OH)Fe(H_2_O)_4_] + 2H_3_O^+^, (2)

[Fe(H_2_O)_6_]^2+^ + H_2_O → [Fe(H_2_O)_5_OH]^+^ + H_3_O^+^ (soluble at pH 8.6). (3)

In the presence of oxidants, e.g., oxygen (in our case trimethylamine-N oxide, (CH_2_)_3_NO), ferrous ions are oxidized to ferric ions, forming insoluble yellow/brown ferric hydroxides in alkaline aqueous solution (solubility of ferric ions could be observed at pH below ~3):

[Fe(H_2_O)_5_OH]^2+^ + H_2_O → [Fe(H_2_O)_4_(OH)_2_]^+^ + H_3_O^+^, (4)

[Fe(H_2_O)(OH)_2_]^+^ + H_2_O → [Fe(H_2_O)_3_(OH)_3_] + H_3_O^+^. (5)

Therefore, a nitrogen atmosphere ensures anaerobic controlled conditions for MFer synthesis. In the presence of a protein cavity (apoferritin), as in the case of ferritin, ferrous ions should first pass through 3-fold protein channels, and are then oxidized and form magnetite during the nucleation and growth of nanocrystals [7]. Two other possible pathways that contribute to the shift of the chemical equilibrium could partially explain the formation of various types of iron compounds, identified in recent magneto-optical (Fe_2_O_3_-like phase) [39] and X-ray studies (*γ*-FeOOH phase) [40]:

Fe^2+^ + H_2_O + O^2−^ (rapid oxidation, 65 °C) → [Fe(H_2_O)_6_]^3+^ → *α*-Fe_2_O_3,_ (6)

Fe^2+^ + H_2_O + O^2−^ → [Fe(H_2_O)_6_]^2+^ / [Fe(H_2_O)_6_]^3+^ (pH < 7; 65 °C) → *γ*-FeOOH. (7)

The first reaction (7) is realized during the preparation of MFer with high iron loadings (LF 1250) [39], when rapid oxidation leads to the most stable iron compound, hematite (*α*-Fe_2_O_3_). The second reaction (8) occurs during the slow oxidation of ferrous ions necessary for the formation of fine magnetite nanoparticles. According to the basic scheme of the synthesis reaction [30], during magnetite synthesis, H^+^ ions are formed in the solution, together with H_2_O_2_ oxidant. These reaction products undesirably change the pH to a lower value and cause uncontrolled additional oxidation of ferrous ions, followed by the formation of lepidocrocite (*γ*-FeOOH) (LF 1294) [40]. This second pathway does not exclude the subsequent formation of magnetite nanoparticles. The addition of NaOH solution during synthesis can shift the chemical equilibrium towards the formation of magnetite at alkaline pH [22,23]. This assessment and the majority of amorphous iron compounds (most likely iron hydroxides/iron-oxo-hydroxides) in sample ^T^MFer (LF 559) rule out the assumption that the iron over-loading effect alone induces magnetite formation. Most likely, another stronger key factor is necessary for biomineralization of magnetite, e.g., high-energy electromagnetic radiation (*γ*-rays). Subsequent mineralization of magnetite in vivo, associated with pathological events, may be a defense mechanism against such energy impact [41].

### 2.4. Protein Structure Variations under Aqueous Medium pH Changes

Firstly, to exclude the variety in the formed iron compounds at a high loading factor, we chose low LF 190 of MFer to study the effect of pH on the MFer structure using small-angle neutron scattering (SANS). The removal of water immediately after the standard synthesis of MFer [30] using lyophilization and storage in the dark protected the material from unwanted thermal or redox transformations of iron cores (by oxygen) or eventually electron transfer induced by natural electromagnetic radiation. The samples were dissolved in a given amount of D_2_O, while the pH was adjusted by adding 0.2 DCl and 2 M NaOD prior to SANS measurements.

**Figure 6:**
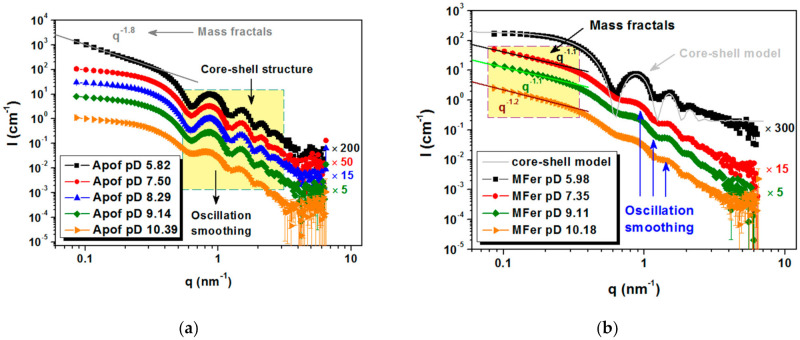
Scattering curves for protein colloidal solutions for: (**a**) apoferritin at full contrast at five different solution pD (10.39; 9.14; 8.29; 7.50 and 5.82) and (**b**) MFer LF 190 at full contrast at five different solution pD (10.18; 9.11; 7.35; and 5.98).

SANS measurements were carried out in the q-range interval from 0.08 to 7 nm^−1^. MFer sample with LF 190 and apoferritin at various pH values at full contrast (in 100% D_2_O) was measured to compare pH-induced structural changes (Figure 6). The measured pH values were converted to pD [42] (negative decimal logarithm of the concentration of deuterium ions in solution) using Equation (9)

pD = 0.929 × pH meter reading + 0.42.(8)

Typical core-shell oscillation peaks were observed at 0.6 nm^−^^1^ for apoferritin in an aqueous medium (Figure 6a). The increased scattering intensity at small q values for pD 5.82 indicated the presence of aggregated particles and the appearance of mass fractals with dimension D = 1.8, modeled using the power-law function I(q)~A/q^α^ [43]. This pD value is near the region of isolelectric point, where apoferritin coagulation at zero total charge is typical (pI ~4.58 ± 0.02) [44]. The smeared oscillations at pD 10.39 are most likely caused by the partial decomposition of the biomacromolecule into protein subunits (Figure 6a).

MFer prepared with relatively low LF 190 was selected as a representative sample to achieve a good core-shell protein structure weakly affected by LF [34]. MFer with the smallest pD ~5.98 close to pI indicated the least stable state according to the charge change, therefore visible flocculation of aggregated objects were separated using centrifugation. After phase separation, the SANS data point to the presence of core-shell structures in the supernatant typical of pure apoferritin (Figure 6b, black curve). This means that part of MFer complexes in solution exhibited no interaction with H^+^ ions under acidic conditions and some apoferritin cages were not able to bind iron and form the MFer complex during synthesis. We assume that aqueous solutions of MFer contain not only MFer particles and their aggregates, but also intact apoferritin molecules. Forward scattering intensities at initial q ~0.08 nm^−1^ taken from black curves (Figure 6a,b) made it possible to compare protein concentration for Apof pD 5.82, i.e., 6 g/L, which was about 12 times higher than calculated for the supernatant of MFer pD 5.98. Apparently, lowering the pH along with the presence of iron can be used to separate intact apoferritin shells from the MFer colloidal solution. In model fitting, the core-shell model was applied using the outer and inner protein radii of 6 and 4 nm, respectively. The real structure obtained by SANS does not fully correspond to the model (Figure 6b, black curves). Deviations from the ideal hollow sphere could related to the inversion of inner or outer amino-acid residues of polypeptide chains or the presence of another internal component, e.g., nucleic acid [45]. This information could explain the autocatalytic character of this flexible and dynamic protein, the function of which is largely dependent on self-assembly [3,4,5,35]. At PD 7.35 and 9.11, the MFer sample was affected predominantly by LF due to the fact that this pD range contributes to neutral stable conditions for this type of protein [46]. On the MFer scattering curve measured at pD 10.18, the oscillations disappear (Figure 6b). It is not possible to determine whether the degree of smearing is induced by LF or a highly alkaline condition. We can only compare it with the behavior of pure apoferritin at pD 10.39 (Figure 6a) and deduce the destabilization of the protein structure dissociated into protein subunits as a typical consequence of alkaline protein hydrolysis. Comparison of the pH-dependent structural behavior (oscillations in the scattering curve) of pure apoferritin (Figure 6a) and MFer with low LF 190 (Figure 6b) clearly shows that iron loading (LF) affects protein structure more noticeably than pH.

### 2.5. SANS Contrast Variation for Iron Core Characterization

**Figure 7:**
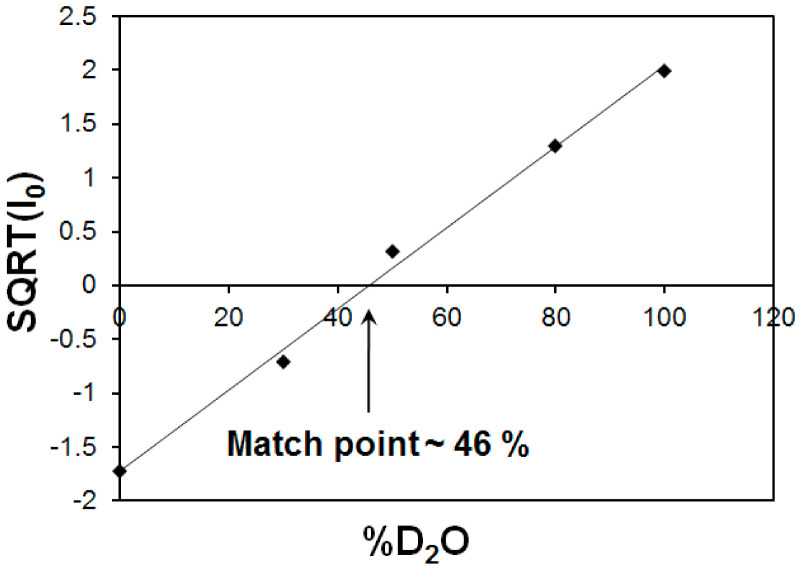
Square root of the scattering intensities at zero angle (I_o0_) as a function of D_2_O volume fraction. The intersection of the fitted straight line with x-axis corresponds to match point of 46% for MFer with LF 190.

The solvent contrast variation method was applied to the MFer sample with LF 190 at a specified pH value of ~7 using 5 different D_2_O/H_2_O ratios in the solvent composition, and protein concentration of ~6 g/L (Figure 7).

The characteristic match point was obtained at 46 % D_2_O, where the scattering length density (SLD) values of the solvent and MFer particles coincide. The SLD of the solvent mixture was calculated using SLD of H_2_O ρ_H2O_ = −0.559 × 10^10^ cm^−2^ and D_2_O ρ_D2O_ = 6.34 × 10^10^ cm^−2^ according to the equation ρ_s_ = ρ_MFER_ = η* ρ_D2O_ + (1 − η)* ρ_H2O_ resulted as 2.60 × 10^10^ cm^−1^, while η = 0.46. The experimental volume fraction, Φ, assuming the presence of magnetite (Fe_3_O_4_) in the core was determined from the equation ρ_s_ = ρ_MFER_ = Φ * ρ_Fe3O4_ + ρ_apof_ * (1 − Φ) is Φ = 0.06; while ρ_Fe3O4_ = 6.91 × 10^10^ cm^−2^ and ρ_apof_ = 2.34 × 10^10^ cm^−2^. The theoretical volume ratio Fe_3_O_4_: apoferritin was found to be 0.006 (using LF-value of 190, number of Fe_3_O_4_ molecules N = 190/3 = 63; M (Fe_3_O_4_) = 231.53 g mol^−1^; N_A_ = 6.022 × 10^23^; ρ_mass_ (Fe_3_O_4_) = 5.15 g cm^−3^ and V (apof) = 744 nm^3^). The volume fraction calculated in the monodisperse approximation obtained from the SANS measurement is therefore about 10 times larger compared to the iron loading in the MFer synthesis. Such a high ratio of the iron compound to the protein indicates a partial destruction of the apoferritin shell [47]. The destruction of the protein cage was most likely caused by a relatively high temperature of synthesis (see explanations to Figure 1) or partially by a slightly alkaline pH of synthesis [34,46], an increase in which usually leads to alkaline hydrolysis of proteins.

### 2.6. Critical Assessment of Synthesis Technology and Application Potential of Magnetoferritin

After extensive long-term research of MFer we summarize here the most important points leading to a shift in its application. To preserve magnetite, we suggest increasing the pH during synthesis by controlled addition of alkali. After synthesis, the MFer solution should be purified (using a combination of magnetic separation, chromatography, and electrophoresis), cooled, and freeze-dried to remove water. Water is an essential medium for the mineralization of nanoparticles in MFer, which accelerates the MFer response to physicochemical changes in the environment. MFer should be stored as a solid powder for applied research, which can increase its shelf life and efficiency. New strategies for nanotechnological design of magnetic-protein superstructures require additional post-synthesis modifications for self-assembly reconstructions.

The contrast variation method in small-angle neutron scattering can help determine the ratio of the iron oxide phase to the protein and determine iron phase variations at the same LF for MFer prepared under different physicochemical conditions. Verification and combining this method with magneto-optical measurements or X-ray diffraction help to characterize samples, which can be useful as a potential in vitro standard contrast agents in MRI diagnostics of various diseases associated with specific content and the type of iron phase formation in the brain or tumors [15,16]. The last review suggests a correlation between the time from a primary trauma event (e.g., brain injury) to death (i.e., agonal state) and an increase in serum ferritin levels. Post-mortem investigation of biochemical markers has its limitations, although it has great prospects for forensic and judicial purposes [48]. Ferritin level and iron core composition determined before and after a traumatic incident could help to estimate survival time and date of injury.

**Figure 8:**
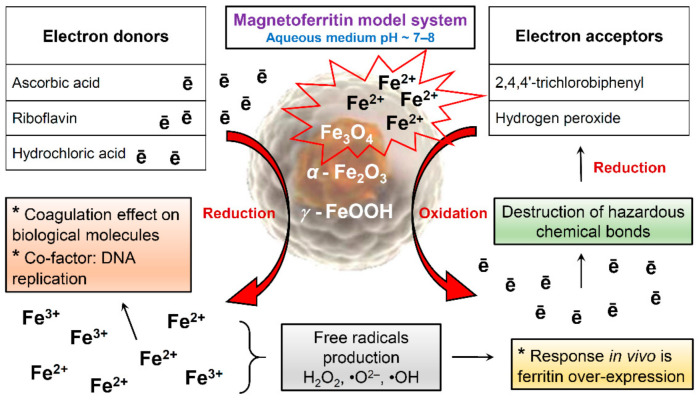
Schematic illustration of magnetoferritin redox reactions during various interactions.

Our studies of samples obtained in vitro do not exclude the simultaneous presence of magnetite, hematite, and lepidocrocite in MFer samples. The iron core in the prepared MFer undergoes post-synthesis transformations associated with redox reactions in response to potential external effects of oxygen, changes in temperature or pH in the liquid state. This apparent lack of sensitivity causes the reactivity of MFer colloidal solutions, especially in interactions with other substances: vitamins [25], dangerous molecules such as polychlorinated biphenyls (PCBs) [49] or hydrogen peroxide (H_2_O_2_) [50], schematically illustrated in Figure 8.

Ascorbic acid and riboflavin, as electron donors, can reduce ferritin and the MFer iron core to ferrous ions [25]. Vitamins can increase the bioavailability of iron for oral iron supplementation-based therapy by fine-tuning the optimal vitamin-to-iron ratio to reduce the harmful gastrointestinal side effects of iron [25,51]. Hydrochloric acid (HCl) can dissolve iron compounds to ferrous and ferric ions, respectively. According to the scheme, the formation of magnetite could serve as a protective measure when interacting with hazardous molecules inside the body, leading to dechlorination of PCBs or decomposition of H_2_O_2_, while the reaction efficiency depends on the loading factor [49,50]. Long-term exposure to synthetically prepared magnetoferritin inside organisms can lead to the loss of magnetic properties and degradation of iron oxides in cells [52]. The biotransformation of the iron oxides used has not yet been clarified. For these reasons, standardized magnetoferritin samples using various physicochemical methods with certain iron loading factors and chemical composition can be very useful as biochemical signal markers for MRI differential non-invasive diagnosis of various diseases or for forensic post-mortem investigation.

The redox reaction in ferritin structures in vivo reports differences reflected in the reduction potential, E_H_, which for ferrihydrite, hematite, and magnetite reactions is: i.e., +0.012 V, −0.286, and −0.310 V, respectively, at pH ~7. The reducibility of iron compounds is a heterogeneous reaction depending on the surface area of iron oxide, aggregate state, and possible surface adsorbates. Redox reactions can affect the pH of the solution, concentration of ferrous iron (Fe^2+^), and the reduction potential of the reductant [53]. Thorough research of various iron compounds in the brain or other organs could help to understand many events, especially in immune responses (e.g., during SARS-CoV-2 virus infection). We assume that studies of the external regulation of oxidation-reduction mechanisms of iron and its compounds inside magnetoferritin in an aqueous environment could help to reveal the useful properties of materials and predict interactions, especially for applied practice.

## 3. Materials and Methods

### 3.1. Chemicals

Chemicals were purchased from Sigma-Aldrich (Saint-Louis, MO, USA) (ammonium ferrous sulphate hexahydrate = Mohr′s salt ((NH_4_)_2_Fe(SO_4_)_2_.6H_2_O), deuterium oxide (D_2_O), equine spleen apoferritin in 0.15 M NaCl, ethanol (C_2_H_6_O), hydrogen peroxide (H_2_O_2_), N-(1,1-Dimethyl-2-hydroxyethyl)-3-amino-2-hydroxypropanesulfonic acid (AMPSO), sodium hydroxide (NaOH), and trimethylamine N-oxide ((CH_2_)_3_NO)), from Fluka (Coomassie brilliant blue), ITES (hydrochlorid acid (HCl)), from Slavus (potassium thiocyanate (KSCN)), and from Centralchem (phosphoric acid (H_3_PO_4_)).

### 3.2. Sample Preparation

First, apoferritin was dispersed in 0.05 AMPSO buffer, adjusted by 2 M NaOH solution to the final pH value of 8.6, and heated to 37, 65, and 75 °C. Then, 0.1 M solution of (NH_4_)_2_Fe(SO_4_)_2_.6H_2_O) and oxidant (0.07 M solution of (CH_2_)_3_NO were added to the reaction mixture in stoichiometric ratio 3:2, 10 times, during 100 min using syringes at a constant temperature of 65 °C with stirring (magnetic stirrer) and heating (IKA C-MAG HS 7). Demineralized water used for all solutions was deaerated using inert nitrogen during ~1 h to ensure anaerobic conditions, then the reaction vial was hermetically closed. Magnetoferritin (MFer) samples with the different loading factor (the average number of iron atoms per one apoferritin molecule) were prepared by varying the amount of the added ferrous ions and oxidant.

### 3.3. Quantitative Ultraviolet and Visible Spectroscopic Analysis of Loading Factor

Quantitative determination of LF was performed using UV-VIS spectrophotometer SPECORD 40 (Analytik Jena, Jena, Germany) at 25 °C with a precision of about 1%. The mass concentration of iron atoms c_m_^Fe^ was determined using the reaction of iron-containing samples with 3% H_2_O_2_ in an acidic medium of concentrated (35%) HCl at 50 °C for 30 min. Fe^3+^ ions produced in the presence of 1M KSCN formed the red thiocyanate complex of Fe[Fe(SCN)_6_], and its light absorbance at the wavelength of 450 nm was measured. The mass concentration of total iron atoms in the sample was calculated using the regression equation from the linear calibration curve. The mass concentration of native apoferritin (NA), c_m_^NA^, was obtained by the standard Bradford method. The absorbance of the blue colored complex of the Bradford agent with the protein residues was detected at the light wavelength of 595 nm after 5 min incubation at 25 °C. From the calculated ratio of c_m_^Fe^: c_m_^NA^ in a given sample volume using the known molecular weights of NA and iron, respectively, LF of MFer was calculated according to the equation:

pD = 0.929 × pH meter reading + 0.42.(9)

In Equation (10), M_NA_ means the molecular weight of native apoferritin (i.e., ~481,200 Da, the value obtained from Sigma-Aldrich), and M_Fe_ represents the standard atomic weight of the iron atom (i.e., ~55.845 Da).

### 3.4. Dynamic Light Scattering from Magnetoferritin

The hydrodynamic diameter for MFer colloidal solutions was determined by dynamic light scattering (DLS) performed at Zetasizer Nano ZS 3600 (Malvern Instruments, Malvern, UK) using the Stokes-Einstein’s equation:

(10) $$D_{T}=\frac{k\;T}{6\;\pi \;\eta \;R_{h}}$$

where *D_T_* is the diffusion coefficient, *k* is the Boltzmann constant, *T* is the temperature, *η* is the solvent viscosity, and *R_h_* is the Stokes, or hydrodynamic, radius of a spherical particle. The hydrodynamic diameter D_HYDR_ was measured using disposable polystyrene cuvettes in the protein mode of the data analysis at 25 °C. The size distribution was displayed in the Zetasizer software as a dependence of the relative number of particles on their size, and the hydrodynamic diameter represented the maximum of the curve. The temperature trend was followed using glass cuvettes in the protein mode of the data analysis.

### 3.5. Colloidal Stability and Determination of the Total Charge

Zeta potential was determined using Laser Doppler velocimetry combined with electrophoresis at Zetasizer Nano ZS 3600 (Malvern Instruments, Malvern, UK) at 25 °C using the Henry’s equation:

(11) $$U_{E}=\frac{2\varepsilon \zeta \;f\left(Ka\right)}{3\eta }$$

where *U_E_* is electrophoretic mobility, which depends on the strength of the electric field or voltage gradient, *ε* is the dielectric constant of the medium, *ζ*-zeta potential, *f(Ka)* is the Henry’s function, and *η* is the medium viscosity. Zeta potential measurements were performed within one minute after filling the folded capillary cells in the auto mode of the data analysis.

### 3.6. Investigation of Morphology by Cryogenic Transmission Electron Microscopy

The morphology and size distribution in the prepared samples were determined by Transmission Electron Microscope Jeol 1400 (JEOL USA, Inc., Peabody, MD, USA) with a resolution of up to 0.2 nm at cryo temperature using cryoTEM holder.

### 3.7. Determination of Magnetic Properties Using SQUID Magnetometry

Magnetic properties of freeze dried MFer powders were investigated using SQUID magnetometer (Quantum Design MPMS 5XL) (Quantum Design, San Diego, CA, USA). The temperature dependences of magnetization (ZFC-FC measurements) were measured in the range of the induction of the magnetic field up to 5 T in the temperature range of 2.0–300 K. The diamagnetic contribution from the sample capsule was subtracted.

### 3.8. Qualitative Determination of Iron Core Composition by High Resolution X-ray Powder Diffraction

High-resolution powder diffraction data were collected on synchrotron beamline I11 (Diamond Light Source, Oxford, UK) using a wavelength of 0.082487 nm at room temperature. The powder was loaded into a 0.7 mm borosilicate glass capillary. High-resolution diffraction data were obtained from the samples using the multi-analyzer crystal (MAC) detectors. The patterns were collected in the 2θ range of 0–150° with 0.001° data step. Analysis of the XRD-diffraction pattern was carried out to evaluate the phase of the alloy using the Match 3 program [54] containing the COD-Inorganics reference database [55]. Samples for HR X-ray diffraction investigation were freeze dried to obtain powders.

### 3.9. Small-Angle Neutron Scattering Structural Analysis

SANS measurements were carried at D22-Large dynamic range small-angle diffractometer at ILL, (Grenoble, France). The multidetector with 16 K resolution elements (128 linear sensitive Reuter-Stokes^®^ detecting ^3^He tubes arranged vertically with a spacing of 8 mm, dead time of 2 μs) moves along the beamline in the vacuum tube providing sample-to-detector distances from 1.1 m to 17.6 m. The covered q-range was from 0.08 to 7 nm^−^^1^. Samples for SANS measurements were first treated using lyophilisation to obtain powders, which are dissolved in the adequate ratio of H_2_O:D_2_O with final c_protein_ of 6 g/L. Possible aggregates, which could contribute to the scattering, were separated using centrifugation.

## 4. Conclusions

This study describes the effect of physicochemical conditions of the surrounding aqueous medium on the size, magnetic properties, composition of the iron core, and protein structure of the ferritin derivate—magnetoferritin. The most relevant parameter is the loading factor. The magnetic properties were strongly affected by the synthesis temperature, which contributed to the formation of aggregates, most likely due to the thermal unfolding of proteins and dipole-dipole interactions of iron cores, which is evidenced by dynamic light scattering. Despite the structural sensitivity of magnetoferritin to the loading factor, synthesis temperature, and pH, our data conclusively confirm the presence of intact core-shell structures in solution after pH lowering. It seems that iron loading along with pH lowering could be a helpful factor for the separation method for MFer segregation from unloaded apoferritin cages in the solution. The comparison of calculation using the theoretical loading factor and scattering length density obtained by contrast variation suggests a high volume ratio of the magnetic phase to the protein. These investigations indirectly point to the dissociation of the protein cage into subunits in solution. The new strategy for nanotechnology design of magnetic-protein superstructures requires the search for additional post-synthesis modifications for self-assembly reconstructions. Despite this, our previous studies have shown that the redox potential of iron phases in the aqueous medium of magnetoferritin opens up a wide range of possibilities in biomedical, forensic, nanotechnology, or environmental practice.

## Figures and Tables

**Table 1:** Summary of measured integral parameters for MFer samples.

Sample LF/T_SYNTHESIS_ (°C)	D_HYDR_ (nm)	PDI	ζ (mV)	M_s_ (A·m^2^·kg^−1^) at 5 T and 22 °C
MFer LF 160/65	18.6 ± 0.7	0.5	−38.4	-
^T^MFer LF 447/75	47.9 ± 0.8	0.4	−27.6	0.025
MFer LF 490/65	19.5 ± 4.3	0.3	−29.2	-
MFer LF 510/65	19.5 ± 1.2	0.3	−37.1	-
^T^MFer LF 559/37	45.6 ± 0.5	0.3	−25.8	0.001
^T^MFer LF 581/65	14.6 ± 2.1	0.3	−26.2	0.028
Ferritin LF 1131	15.6 ± 1.2	0.3	−21.2	0.003
Apoferritin	11.9 ± 0.4	0.4	−23.1	-

LF represents loading factor, T_SYNTHESIS_ (°C) synthesis temperature, D_HYDR_ (nm) hydrodynamic diameter, PDI polydispersity index, *ζ* (mV) zeta potential, M_s_ (A·m^2^·kg^−^^1^) magnetization saturation at magnetic field 5 T measured at 22 °C. T prefix in ^T^MFer labeling was used for specification of MFer samples prepared at three different synthesis temperatures with similar LF for magnetic and structural comparative studies.

## Data Availability

The data presented in this study are available on request from the authors.

## Sample Availability

Not available.
